# Vibration-Based SHM in the Synthetic Mooring Lines of the Semisubmersible OO-Star Wind Floater under Varying Environmental and Operational Conditions

**DOI:** 10.3390/s24020543

**Published:** 2024-01-15

**Authors:** Nikolas P. Anastasiadis, Christos S. Sakaris, Rune Schlanbusch, John S. Sakellariou

**Affiliations:** 1 Norwegian Research Centre, Technology Department, Jon Lilletuns vei 9 H, 3. et, 4879 Grimstad, Norway; nikolas.p.anastasiadis@gmail.com (N.P.A.); csak@norceresearch.no (C.S.S.); 2 Department of Mechanical Engineering and Aeronautic, University of Patras, 26504 Patras, Greece; sakj@mech.upatras.gr

**Keywords:** structural health monitoring, synthetic fiber ropes, varying environmental and operating conditions, transmittance function, autoregressive models, Floating Offshore Wind Turbine, mooring lines, damage detection, functional models

## Abstract

As the industry transitions toward Floating Offshore Wind Turbines (FOWT) in greater depths, conventional chain mooring lines become impractical, prompting the adoption of synthetic fiber ropes. Despite their advantages, these mooring lines present challenges in inspection due to their exterior jacket, which prevents visual assessment. The current study focuses on vibration-based Structural Health Monitoring (SHM) in FOWT synthetic mooring lines under uncertainty arising from varying Environmental and Operational Conditions (EOCs). Six damage detection methods are assessed, utilizing either multiple models or a single functional model. The methods are based on Vector Autoregressive (VAR) or Transmittance Function Autoregressive with exogenous input (TF-ARX) models. All methods are evaluated through a Monte Carlo study involving 1100 simulations, utilizing acceleration signals generated from a finite element model of the OO-Star Wind Floater Semi 10 MW wind turbine. With signals from only two measuring positions, the methods demonstrate excellent results, detecting the stiffness reduction of a mooring line at levels 10% through 50%. The methods are also tested for healthy cases, with those utilizing TF-ARX models achieving zero false alarms, even for EOCs not encountered in the training data.

## 1. Introduction

### 1.1. The General Problem

The offshore wind energy sector is undoubtedly undergoing unprecedented growth, with projections indicating substantial expansion in the coming years. As nations strive to reduce carbon emissions, offshore wind power has emerged as an essential solution for coastal countries. The European Union has set new targets, aiming to achieve 111 GW offshore wind capacity by 2030 and a staggering 317 GW by 2050, surpassing the already ambitious goals of 60 and 300 GW set in November 2020 [1]. Similarly, the United States of America has established policies with the goal of installing 30 GW of offshore wind power by 2030 [2], while within Asia, China alone targets 200 GW by 2030 [3]. For the effective achievement of these ambitious targets and the alignment with the global goals for net-zero emissions by 2050, the acceleration of the annual deployment rate of offshore wind projects is crucial [4]. Yet, the offshore wind industry faces significant challenges, which include not only the total lifecycle costs of the turbines themselves but also the inherent risks associated with these substantial investments.

The operational and maintenance (O&M) cost plays a pivotal role in the overall offshore wind turbine life expenses, comprising a range of 13.9% to 19.6% of the levelized cost of energy (LCOE) [5,6]. Among these costs, the maintenance strategy itself is a major factor. Evidently, the utilization of corrective maintenance, which involves addressing issues as they occur, is not a sustainable approach for offshore wind turbines due to the substantial ramifications of prolonged downtimes [7]. Preventive maintenance schemes, which depend on frequent inspections aimed at addressing issues before they lead to failure, hold the potential to mitigate these maintenance-related downtimes; however, the harsh oceanic conditions limit the available time windows for on-site inspections, extending the periods between them. Consequently, more time is allowed for damage to develop into an irreparable level before being detected [7]. Moreover, planned on-site inspections demand considerable downtime, as many of them cannot be conducted while the turbine is operational. Finally, given that floating wind turbines can be situated well over a hundred kilometers from the shore (e.g., the Hywind Tampen wind farm located in the Norwegian North Sea [8]), the transportation cost for the inspections is also substantial. These challenges can be mitigated with condition-based maintenance strategies, which involve the continuous remote measurement of relevant information regarding the structural state, as well as the utilization of Structural Health Monitoring (SHM) techniques for damage detection [9].

A critical part of Floating Offshore Wind Turbines (FOWTs) that requires monitoring is the mooring lines, as their failure may lead to several catastrophic events [10]. Firstly, such failure will result in a drift, disconnecting the electric cable, jeopardizing the power production, and introducing the risk of collision with other wind turbines. Furthermore, the stability of the structure itself can be compromised, resulting in increased oscillations, subjecting other critical components to additional stress [11], and potentially leading to capsizing or even sinking. It becomes evident that maintaining an up-to-date understanding of the mooring lines’ condition is essential for preventing these dire consequences.

When it comes to deep-sea installations, conventional chain or steel wire are not appropriate options for the mooring system since, with increasing depth, the weight of the mooring line alone exceeds its stress limit. Synthetic fiber ropes are advantageous in this regard, as their density is comparable to water’s, allowing for neutral buoyancy and, therefore, rendering them suitable for any depth [9]. Furthermore, they possess greater fatigue and corrosion resistance, which are significant design factors in the corrosive and dynamically loaded offshore environment [12]. Nonetheless, these advantages come with some challenges when it comes to monitoring the lines.

**Figure 1:**
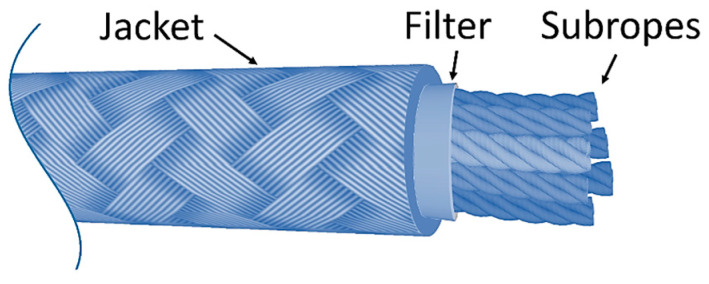
Parallel sub-rope mooring line composition.

Usually, mooring lines are monitored through visual inspections using Remote Operated Vehicles (ROVs). Although this approach may be suitable for monitoring chains or steel wires, it is not for synthetic mooring lines. This is because the complex composition of synthetic ropes and their jacket, if present, hinders potential damage in the internal strands [13] (see Figure 1). Furthermore, biofouling and murky water might also obstruct reliable visual inspection. Therefore, alternative methods of monitoring must be employed.

For the monitoring of synthetic mooring lines, vibration-based methods constitute a fitting choice due to their several advantages [14]. Firstly, they possess the potential to detect a wide range of damage, including those that may not be visible from the outside of the jacket. Additionally, these methods are cost-effective in terms of instrumentation and operation, as they require only a small number of sensors. The sensors, typically accelerometers, can be easily placed on the jacket’s exterior, avoiding interference with the rope’s structure and its properties, unlike embedded technologies (e.g., optical fibers) [15]. Furthermore, they do not require the presence of personnel on the wind farm, enabling real-time inspection and reducing transportation costs and occupational risks. Finally, these methods can be extended for damage identification, quantification, localization, and estimation of the remaining service life of the rope, depending on the availability of signals from damaged cases.

Despite their advantages, vibration-based methods do present some challenges, with the most prominent one being their sensitivity to uncertainties, particularly those posed by varying Environmental and Operational Conditions (EOCs). This sensitivity may result in a high rate of false alarms, rendering them impractical.

As of now, the existing literature on vibration-based SHM for mooring lines in FOWTs is very limited. In their work, Gorostidi et al. [16] employ a deep Neural Network (NN) method to detect an increase in the mass of the mooring lines of a semisubmersible FOWT due to biofouling. Jamalkia et al. [17] propose a fuzzy logic-based method for the detection, identification, and quantification of stiffness reduction in the mooring lines of a Tension Leg Platform (TLP) and Spar FOWT. Dehkharghani et al. [18] introduce a nonprobabilistic method utilizing artificial NNs to detect and identify stiffness reduction in the mooring lines of a semisubmersible FOWT. Liu et al. [19] demonstrate a mixed model and signal-based approach for the detection of faults on a triple-spar FOWT, including damage on the fairlead and anchor of a mooring line.

While the aforementioned studies make valuable contributions to the field, it is essential to note that they do not adequately address the uncertainties stemming from EOCs. For example, Jamalkia et al. [17] introduced a signal-to-noise ratio to the method’s extracted features, but they only considered a single case of EOCs with constant wind velocity and sinusoidal wave excitation. Dehkharghani et al. [18] introduced some uncertainty due to the stochasticity of the wind and wave excitations, but similar to Jamalkia et al. [17], only accounted for one case of EOCs. Liu et al. [19] considered both laminar wind and stochastic wind and wave excitations, with four cases of EOCs. Although their results are promising in detecting mooring line damage, the limited cases of EOCs used in their evaluation led to the acknowledgment that further investigations are necessary to prove the method’s robustness. Finally, Gorostidi et al. [16] used a large number of different EOCs, with stochastic wind and wave excitation, for the training and evaluation of their method. However, their deep NN approach demands thousands of training cases, which raises questions regarding its feasibility from a practical perspective.

Drawing upon the aforementioned references, the primary objective of the present study is to propose and evaluate damage detection methods that effectively account for uncertainties arising from EOCs. The evaluation of these methods will be characterized by a comprehensive consideration of uncertainties arising from diverse EOCs while ensuring their practicality through economical utilization of available data.

### 1.2. Conceptual Approach: Mitigating Uncertainties in Vibration-Based SHM

Vibration-based Structural Health Monitoring (SHM) methods can be either physics-based or data-driven. Physics-based methods, although they allow for a more thorough understanding of how specific damage may affect the structure’s response, require physics-based models with sufficient precision, which are often not available [14] (pp. 154–156). On the other hand, data-driven methods do not require such models and are, therefore, deemed more appropriate for the current application.

Data-driven vibration-based methods consist of two phases: the training or baseline phase and the inspection phase. In the training phase, while the structure is in the healthy state, signals are obtained and used to estimate one or more baseline models that represent the healthy structural dynamics. During the inspection phase, when the structure’s state is unknown, new signals are acquired and used to estimate a model that represents the dynamics of the unknown-state structure. A significant deviation between the unknown-state and baseline models signifies an alteration in structural dynamics, presumed to be a result of damage.

There are several types of models that are used for the representation of the structural dynamics [20,21,22] (pp. 79–139). The use of parametric stochastic models of the Autoregressive–Moving Average with exogenous input (ARMAX) family is a popular choice due to their formulation, which allows their parameters to be directly associated with the dynamic characteristics of the considered structure (i.e., natural frequencies and damping ratios). This marks a notable distinction from methods that employ data to tune artificial NN parameters, which lack physical interpretability [16,18].

Vibration-based methods can be classified into input–output or output-only, depending on the availability of the excitation signal. Input–output methods usually rely on the deliberate excitation of the structure using mechanical actuators, enabling better control over the excitation frequency bandwidth and energy. Therefore, the obtained vibration response(s) provide detailed information about the structural dynamics across the entire selected spectrum, leading to a more accurate evaluation of the structural state compared to output-only methods. Such methods may utilize ARX models, which, during their estimation, in addition to the measured vibration response(s), incorporate the excitation, thereby enhancing their resilience against excitation variability [21,22] (pp. 81–83). Conversely, output-only methods depend exclusively on vibration response signals obtained under ambient and unknown excitation. This makes them suitable for scenarios where employing a mechanical actuator is either costly or impractical. Nevertheless, the compromise for this practical convenience is the limitation of frequency content in the measured vibration signals, constraining the exploration of structural dynamics. This constraint primarily arises from the bandwidth of the excitation, which is limited to lower frequencies, especially when physical excitations like wind and waves are considered.

To mitigate the impact of excitation variability on the estimated models, which might otherwise compromise the method’s performance, while adhering to response-only type methods, the use of the Transmittance Function (TF) is a viable approach [23]. The TF is defined as the ratio of the Cross-Spectral Density (CSD) over the Power (auto)-Spectral Density (PSD) of two response signals, $y_{1}\left[t\right]$ and $y_{2}\left[t\right]$, measured at different positions on the structure [23]:

(1) $$T\left(j\omega \right)=\frac{S_{y_{2}y_{1}}\left(j\omega \right)}{S_{y_{1}y_{1}}\left(\omega \right)}$$

where ω designates frequency, j the imaginary unit, $S_{y_{2}y_{1}}$ the CSD of $y_{1}\left[t\right]$ and $y_{2}\left[t\right]$, and $S_{y_{1}y_{1}}$ the PSD of $y_{1}\left[t\right]$.

**Figure 2:**
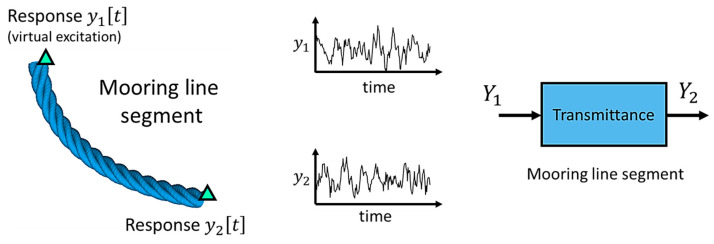
Mooring line segment’s modeling based on the TF of two acceleration signals. 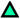: accelerometer measuring position.

The TF resembles a typical Frequency Response Function (FRF), with the distinction that two separate output signals are used instead of a pair of input–output signals. To facilitate a better understanding of the TF functionality within the specific context of the current study, Figure 2 illustrates its application in modeling a mooring line segment. As depicted in the figure, two response signals are measured using accelerometers, one at each side of the segment. By designating one of the two measured signals as the excitation and the other as a response, it becomes possible to define a system that represents the relative admittance between the two measuring positions. The underlying idea for its application is that akin to a conventional FRF, the TF remains unaffected by the excitation variability while exhibiting high sensitivity to damage.

The TF similarity with the FRF also allows for the parametric ARX (TF-ARX) representation of the TF [23], providing a different approach when multiple signals are available, in addition to the Vector-AR (VAR) stochastic models [24,25].

EOCs play a significant role in system dynamics. A conventional data-driven model of any of the ARMA, Vector ARMA, (VARMA) and ARMAX types can represent the healthy structural state under a single EOC. Hence, an alternative approach is essential to accommodate variations in EOCs and prevent their misinterpretation as damage. This approach may be grounded in two main concepts. The first involves the use of implicit methods, such as the Factor Analysis (FA) [26] and Principal Components Analysis (PCA)-based methods [27], which extract features from the vibration signals that are exclusively sensitive to damage and not to the EOCs. The second concept involves the use of explicit methods such as the Multiple Model (MM)-based methods [27,28] and the Functional Model (FM)-based methods [29,30], where the influence of the EOCs is incorporated in the models representing the healthy structural dynamics.

### 1.3. Aim and Objectives

Building on the literature referenced in Section 1.1, the primary aim of this study is to propose and evaluate methods that successfully address uncertainties stemming from EOCs, which may otherwise lead to unreliable damage detection. The proposed methods adhere to either implicit and/or explicit approaches while upholding practicality through the economical utilization of available data. In particular, six methods are employed:

1. Two Multiple Model (MM)-based methods;
2. Two PCA-based MM methods;
3. Two Functional Model (FM)-based methods.

The employed methods are based on either VAR or TF-ARX models and are abbreviated as MM-VAR, MM-TF-ARX, PCA-MM-VAR, PCA-MM-TF-ARX, FM-VAR and FM-TF-ARX.

The methods are evaluated and compared through 1100 Monte Carlo simulations utilizing acceleration signals generated from the simulation of the OO-Star Wind Floater Semi 10 MW FOWT. Several damage scenarios are considered corresponding to 10% to 50% stiffness reduction of a single mooring line. Damage detection is based on acceleration signals from a limited number of measuring positions, with the optimum positions selected via criteria based on the PSD and the TF.

The remainder of the article is organized as follows: Section 2 provides an overview of the FOWT, the Monte Carlo simulations, and a comparative analysis of the changes in the structural dynamics resulting from damage in a mooring line and changes in EOCs. Additionally, this section presents a systematic approach for selecting the optimal measuring positions for damage detection based on specific criteria. In Section 3 the MM, PCA-based MM, and FM-based methods’ framework is introduced, along with the models employed within them. Section 4 provides the system identification process and the results of the detection methods. These results are subsequently reviewed in Section 5, and conclusions are provided in Section 6. Preliminary results of this study based on scalar models have been presented in a conference paper [31].

## 2. Case Study

### 2.1. The OO-Star Wind Floater Semi 10 MW FOWT

**Figure 3:**
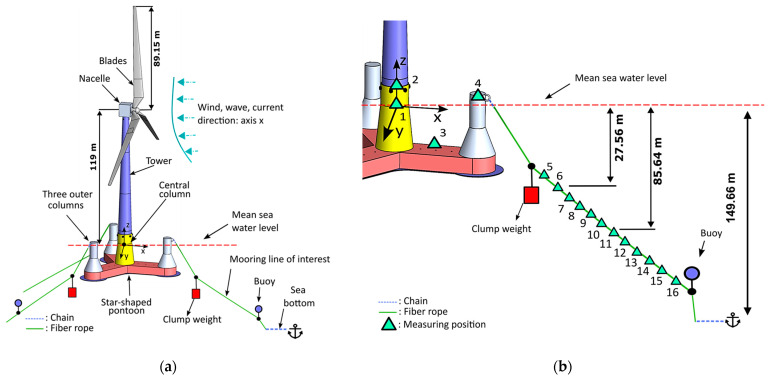
(**a**) The OO-Star Wind Floater Semi 10 MW FOWT and (**b**) the measuring positions on the synthetic fiber rope and the floater. The numerical labels alongside the measuring position symbols represent the corresponding measuring positions 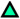.

The case study for the implemented methods involves a simulation model of a 10 MW Reference Wind Turbine developed at the Technical University of Denmark (DTU) [32] mounted on the OO-Star Wind Floater Semisubmersible substructure, which is designed by Dr. Techn. Olav Olsen AS (DOOA) [33,34]. As illustrated in Figure 3a, the substructure is composed of four columns, three outer and one central, interconnected with a star-shaped pontoon. The central column provides support for the wind turbine, while the outer columns contribute to the overall stability of the structure. The entire substructure is constructed from posttensioned concrete and is secured in position using a semitaut mooring system consisting of three lines attached to the top section of the outer columns. Each mooring line is primarily composed of synthetic rope except for the bottom part, which is in contact with the seabed, and a small segment at the connection with the substructure, both of which are composed of steel chains. At a specific point on each mooring line close to the surface, a clump weight is placed to enhance the substructure’s restoring forces, thereby reducing its vertical offset. Furthermore, a buoy is attached to each line, positioned between the synthetic rope and the steel chain segments, which has the primary function of preventing the synthetic rope from making direct contact with the seabed.

The examined substructure presents several advantages, which makes it a compelling option for renewable energy production. Firstly, the semisubmersible substructure can be manufactured and assembled alongside the wind turbine off-site and then towed to its designated location, avoiding the additional costs and risks associated with complex on-site installation. In the case of utility-scale wind turbines, the transportation of their components at the assembly location is also a significant concern. Concrete as a material is advantageous in this regard because it can be easily transported directly to the construction site in its raw form [35]. Furthermore, it has higher local availability than steel, given that most countries have one or several cement producers. Additionally, concrete has a lower cost than steel while also exhibiting higher corrosion resistance, lower operation and maintenance costs, and a longer design life [36].

The OO-Star floater has been investigated extensively within the LIFES50+ [33,37] and FLAGSHIP [34] projects and is now owned by Bouygues Travaux Publics.

### 2.2. The Monte Carlo Simulations

The vibration signals used for the evaluation of the implemented SHM methods were obtained from Monte Carlo simulations conducted through the aero-hydro-servo-elastic simulation tool 3DFloat, developed by the Institute of Energy and Technology (IFE) [38]. This tool uses a Finite Element Model (FEM) with 2706 Degrees of Freedom, created jointly by DOOA and IFE. During the Monte Carlo simulations, the operating wind turbine was subjected to stochastic wind and wave excitations. The wind was generated following the Kaimal spectrum model [39] (p. 30), while the waves followed the JONSWAP spectrum [40] (pp. 106–112). The determination of the structure’s nonlinear response with 3DFloat is based on a corotated FEM approach [41]. In this approach, the element equations are formulated at each time step in a coordinate system attached to a reference configuration, which represents a deformed state of the previous time step. This method enables the use of small-strain elements to accommodate significant global deflections, thereby accounting for geometric nonlinearities. Mooring lines are simulated as Euler–Bernoulli beam finite elements, considering both extensional and bending stiffness. The incorporation of bending stiffness in the model is well suited for fiber rope mooring lines, in contrast to alternative models that account for only extensional stiffness, making them suitable exclusively for chains. The outputs of 3DFloat may include motions, stresses, and other types of signals regarding the operating system.

The SHM methods evaluated in the present study make use of acceleration signals. Multiple measuring positions were considered both along the mooring line and on the substructure, as depicted in Figure 3b. At each position, three signals were generated, corresponding to the x, y and z directions. A sampling frequency of $f_{s}=$ 5 Hz was selected, considering the low-frequency wind and wave excitations [37]. Details regarding the simulated cases are shown in Table 1 and discussed in Section 2.3, Section 2.4 and Section 2.5.

### 2.3. The Damage Scenarios

Throughout their lifespan, synthetic rope mooring lines are vulnerable to several damage modes that require attentive monitoring [13,15]. During regular operation, the rope experiences cyclic loading, which, over time, results in tensile and compression fatigue. The primary mechanism of tensile fatigue is the internal fiber-on-fiber abrasion caused by the rubbing of strands within the rope (see Figure 1). This continuous friction eventually leads to the breaking of the rope’s yarns. Compression fatigue, on the other hand, happens when the strands buckle or kink under compression, resulting in a reduction of the fibers’ strength and allowing for increased interyarn movement, which, in turn, leads to more abrasion [15]. Overloading or shock due to extreme weather conditions, ship or trawl collisions and seismic events are also relevant damage modes that can result in significant strength loss or even fracture. Finally, when a rope is subjected to constant load over a long period, its length is increased, a phenomenon also known as creep. If the period is long enough, this elongation becomes irreversible, altering the molecular polymer chains and eventually leading to an increase in stress that accelerates into failure [15].

The discussed damage modes depend on the stress and/or deformation profile of the line. Cyclic loading occurs throughout the entire length of the rope, although certain regions that experience higher load amplitudes are expected to be more affected by tensile fatigue. Regions that operate in lower tension during the alternate loading are, on the other hand, more likely to buckle and thereby undergo compression fatigue, while sections subjected to constant static loading are more likely to undergo substantial creep, depending on the fiber material [15]. The determination of the degradation’s distribution along the line’s length is a challenging task. It depends not only on the spatial and temporal stress profile of the line but also on the influence of each damage mechanism, the quantification of which is itself dependent on many factors. However, such an analysis falls out of the scope of this study, and uniform degradation was adopted herein as in Jamalkia et al. [17] and Dehkharghani et al. [18]. In particular, nine scenarios of different types of damage were considered, corresponding to stiffness reduction from 10% to 50% (refer to Table 1) along the entire length of one of the synthetic fiber ropes (see Figure 3a).

It is important to note that one of the major challenges associated with synthetic mooring lines is their handling during installation, as synthetic ropes are susceptible to damage when mishandled by contractors operating on tight time schedules. Although identifying initial weaknesses in the mooring systems is also of significant interest, this study exclusively focuses on damage that might occur in the ropes after an initial period of operation. Therefore, the assumption is made that during this initial period, the mooring lines remain in a healthy state.

### 2.4. The Environmental and Operational Conditions

As stated before, the EOCs have a significant impact on the structural dynamics. Wind speed, direction, wave height, temperature, and other environmental factors are important sources of uncertainty that should be considered when assessing the robustness of vibration-based SHM methods. In the current study, uncertainty was introduced to the dynamics by considering different mean wind speed values ${\bar{U}}_{hub}$, measured at hub height (see Figure 3a), within the 7–12 m/s range. The significant wave height $H_{s}$ and peak period $T_{p}$ were also treated as variables, dependent directly on the wind speed, as shown in Table 2. The considered scenarios correspond to normal weather conditions, in which the FOWT is in operation, and not to extreme events.

### 2.5. Influence of the EOCs and Damage on the Structural Dynamics

**Figure 4:**
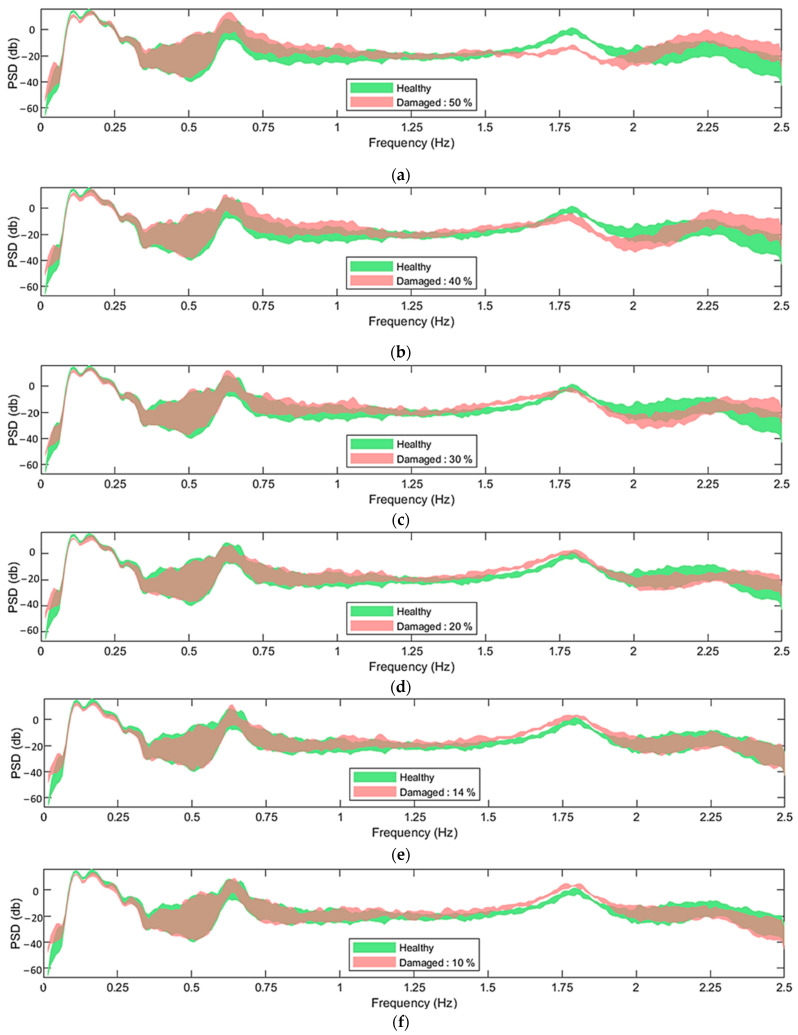
Welchbased estimates of the PSDs of acceleration signals corresponding to measuring position 11 in the x direction, under varying ${\bar{U}}_{hub}\;\in \left[7,\;12\right]$ m/s and corresponding $H_{s}$, $T_{p}$ (refer to Table 2), for healthy and damaged states: (**a**) damage level 50%; (**b**) damage level 40%; (**c**) damage level 30%; (**d**) damage level 20%; (**e**) damage level 14%; (**f**) damage level 10%. For the Welch estimate: Hamming Window; window size: 350; $f_{s}\;$= 5 Hz; overlap: 95%; sample size N=8500.

**Figure 5:**
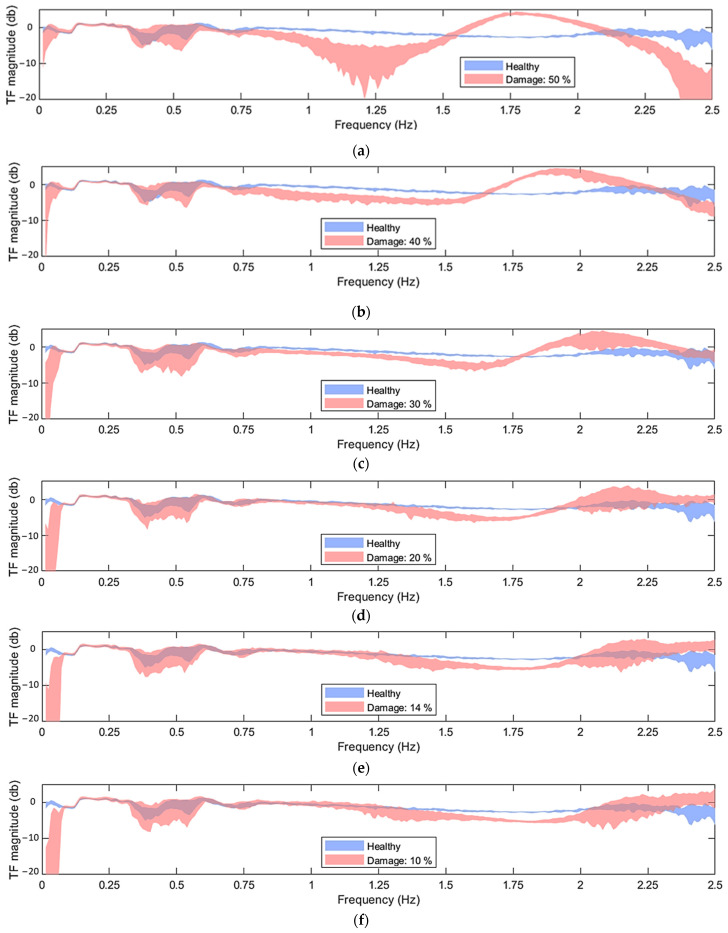
Welchbased TF of acceleration signals corresponding to measuring positions 10 and 9 in the x direction under ${\bar{U}}_{hub}\;\in \left[7,\;12\right]$ m/s and corresponding $H_{s}$, $T_{p}$ (refer to Table 2), in the healthy and damaged states: (**a**) damage level 50%; (**b**) damage level 40%; (**c**) damage level 30%; (**d**) damage level 20%; (**e**) damage level 14%; (**f**) damage level 10%. For the Welch estimate: Hamming Window; window size: 350; $f_{s}\;$= 5 Hz; sample size N=8500; overlap: 95%.

The challenge addressed in the current study is demonstrated by comparing the effects of EOCs on the FOWT dynamics with those of the induced damage. This comparison is illustrated in Figure 4 and Figure 5 through the nonparametric PSD and TF estimates of the acceleration signals corresponding to indicative measuring positions. Specifically, Figure 4 illustrates the PSD corresponding to position 11 of the mooring line in the x direction, while Figure 5 the TF corresponding to positions 9 and 10 in the x direction as well (see Figure 3b).

In Figure 4, the green areas correspond to the healthy state under the EOCs specified in Table 2, while the red areas correspond to the damaged states with damage levels of {50, 40, 30, 20, 14 and 10} %, also under varying EOCs. The challenge in the damage detection problem lies in the difficulty of distinguishing whether the measured dynamics of a signal under an unknown state reside in the healthy (green area) or the damaged state (red area). As depicted in the figure, the complexity of this task escalates substantially as the examined damage levels decrease. Specifically, in Figure 4a,b, the introduction of a 50% and 40% stiffness reduction, indicative of major damage, significantly influences the structural dynamics within the frequency range of [1.5–2.5] Hz, facilitating a more pronounced differentiation between the healthy and damaged areas. Stiffness reductions of 30% and 20% (shown in Figure 4c,d), representative of moderate damage levels, introduce a more subtle impact on the structural dynamics; nevertheless, in these instances, the two areas are discernible as well. Finally, instances characterized by 10% and 14% stiffness reduction (depicted in Figure 4e,f), indicative of minor damage, yield a negligible effect on the structural dynamics. Consequently, the healthy and damaged regions become nearly indistinguishable.

The PSD serves as a reflection of the frequency contents present in the measured vibration signals. It encapsulates information regarding the structural dynamics across the frequency bandwidth induced by the wind and waves and will, therefore, exhibit high sensitivity to uncertainties stemming from these ambient excitations. As previously stated, the TF may exhibit reduced sensitivity to such uncertainties, enhancing the resilience of the damage detection methods.

The use of TF necessitates good coherence between the employed signals, with values close to unity, indicating low signal-to-noise ratio and measurement errors as well as weak nonlinear or time-variant behavior of the structure [42] (pp. 20, 135):

(2) $$C\left(\omega \right)=\frac{{\left|S_{y_{2}y_{1}}\left(j\omega \right)\right|}^{2}}{S_{y_{1}y_{1}}\left(\omega \right)S_{y_{2}y_{2}}\left(\omega \right)}\;$$

**Figure 6:**
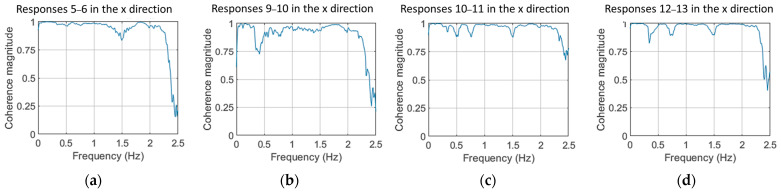
Coherence function of acceleration signals in the x direction of measuring positions: (**a**) 5–6; (**b**) 9–10; (**c**) 10–11 and (**d**) 12–13. For the Welch estimate: Hamming Window; window size: 350; $f_{s}\;$= 5 Hz; overlap: 95%; Sample size N=8500. Shown for a case under ${\bar{U}}_{hub}=10$ m/s.

To verify that the TF can be used, the coherence function was assessed for each possible combination of measuring positions. The position combinations with the best coherence function values are shown in Figure 6. It is worth noting that a relatively good coherence was observed only among responses in the x direction and between adjacent measuring positions, excluding pairs 1–2, 2–3, 3–4, 4–5 and 15–16. This may be attributed to the wind and wave directions in relation to the mooring line topology (see Figure 3a) and the closeness of the adjacent measuring positions (Figure 3b). As shown in Figure 3a, the mooring line under examination extends in the x-direction, which is aligned with the wind and wave propagation directions. This alignment might result in heightened vibrations in this specific direction. For neighboring sensor positions in the x direction, the coherence demonstrated favorable values, except within the frequency range of [2.25–2.5] Hz.

Based on Figure 5, and in comparison to the PSD shown in Figure 4, it becomes evident that the TF allows for a clearer distinction between healthy and damaged cases, even in instances of slight damage (see Figure 5e,f).

**Figure 7:**
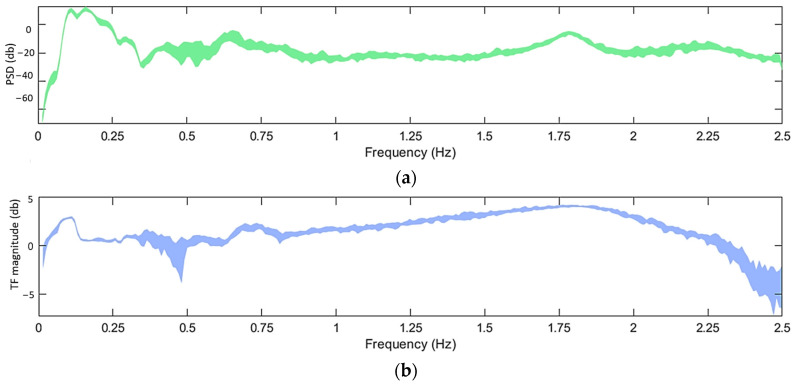
(**a**) Welchbased PSD of acceleration signals corresponding to measuring position 11 in the x direction and (**b**) Welch-based TF of acceleration signals corresponding to positions 10 and 9 in the x direction for realizations with different random seeds under ${\bar{U}}_{hub}=10\;m$/s. For the Welch estimate: Hamming Window; window size: 350; $f_{s}$ = 5 Hz; sample size N=8500; overlap: 95%.

In addition to the variability of ${\bar{U}}_{hub}$, $H_{s}$, and $T_{p}$, the stochastic nature of wind and wave excitations is also a source of uncertainty. In this simulated model, this uncertainty was addressed by employing various random seeds in the pseudorandom generation of the wind and wave excitations. The influence of this uncertainty is highlighted in Figure 7 where the PSD and the TF are depicted for a specific scenario of EOCs but using different realizations. In the context of this study, the stochasticity of the wind and wave excitations was taken into account in the SHM methods by utilizing multiple signals which were produced during the simulations using different random seeds.

### 2.6. Selection of the Measuring Positions

One of the key considerations for selecting SHM methods is the instrumentation and installation cost. In this context, vibration-based methods have an advantage as they require a relatively small number of sensors when they are strategically placed. In the current study, the optimal measuring positions were determined by evaluating the sensitivity of the response signals’ PSD and TF to damage. This was achieved through the Frequency Response Assurance Criterion (FRAC), the Frequency Amplitude Assurance Criterion (FAAC) and the average Local Amplitude Criterion ($\bar{LAC}$) [29]; vectors are indicated throughout this paper by lowercase boldface, and matrices by uppercase boldface symbols.

(3) $$FRAC=\frac{{\left|h_{o}^{T}h_{d}\right|}^{2}}{\left(h_{o}^{T}h_{o}\right)\left(h_{d}^{T}h_{d}\right)}$$

(4) $$FAAC=\frac{2\left|h_{o}^{T}h_{d}\right|}{\left(h_{o}^{T}h_{o}\right)+\left(h_{d}^{T}h_{d}\right)}$$

(5) $$\bar{LAC}=\frac{1}{n_{\omega }}\overset{n_{\omega }}{\underset{i=1}{\sum }}\frac{2\left|H_{o}^{*}\left(j\omega_{i}\right)H_{d}\left(j\omega_{i}\right)\right|}{H_{o}^{*}\left(j\omega_{i}\right)H_{o}\left(j\omega_{i}\right)+H_{d}^{*}\left(j\omega_{i}\right)H_{d}\left(j\omega_{i}\right)}\;$$

with o and d the subscripts designating the healthy and damaged states, respectively, T and ∗ as superscripts indicating matrix or vector transpose and complex conjugation respectively, $\left|\cdot \right|$ the magnitude of a complex quantity, $H\left(j\omega_{i}\right)$ the PSD or TF at frequency $\omega_{i}$, h a vector consisting of the PSD or TF for successive (discrete) frequencies and $n_{\omega }$ the number of the employed discrete frequency values. The values of these criteria serve as indicators of sensitivity to damage, with the most sensitive measuring positions being those that yield the FRAC, FAAC, and $\bar{LAC}$ values with the highest deviation from the unit. From the above equations, each value of the FRAC, FAAC and $\bar{LAC}$ was calculated using the PSDs or TFs of the signals corresponding to individual pairs of healthy $h_{o}$ and damaged $h_{d}$ state cases of the same EOCs.

In the current study, to account for the various cases of EOCs and the different damage levels, the criteria were calculated for multiple pairs of $h_{o}$ and $h_{d}$. The mean values of these criteria were then employed to determine the optimal measuring position. Specifically, for each ${\bar{U}}_{hub}$ from the set {7, 8, 9, 10, 11, 12} m/s, five criteria values were computed. These values correspond to pairs of healthy and damaged cases, with damage magnitudes of {10, 20, 30, 40  and 50} %. As a result, the total number of $h_{o}$ and $h_{d}$ pairs was 30, which means that 30 values were computed for each measuring position (or measuring position couples when the TF is used). It is worth noting that the excitation in the healthy and damaged cases of each signal was generated using distinct random seeds to account for uncertainty arising from the wind and wave stochasticity.

In Figure 4 and Figure 5, it can be observed that for frequencies under 1 Hz, the PSD and TF are generally highly sensitive to the varying EOCs, while the difference between the healthy and damaged areas is not significant. For this reason, it was decided to exclude the [0, 1] Hz frequency bandwidth for the criteria calculation. As discussed in Section 2.5, the use of the TF requires coherence values close to one. Hence, measuring position pairs with low coherence were excluded from consideration. Additionally, since all measuring position pairs exhibited low coherence values beyond 2.25 Hz, frequencies in the range of $\left[2.25,\;2.5\right]\;Hz$ were also excluded from the criteria calculation when the TF was used. In summary, the frequency bandwidths that were used for the criteria calculations based on the PSD and TF are $\left[1,\;2.5\right]\;Hz$ and $\left[1,\;2.25\right]\;Hz$, respectively.

For the PSD and TF estimation, the Welch method was applied, utilizing a Hamming window with a window size of 350 samples and an overlap of 95%. The signals used had a length of N=8500 samples and a sampling frequency of $f_{s}=5\;Hz$. The achieved frequency resolution is δf=0.0143 Hz, resulting in $n_{\omega }=104$ PSD and $n_{\omega }=88$ TF values for the criteria calculation.

**Figure 8:**
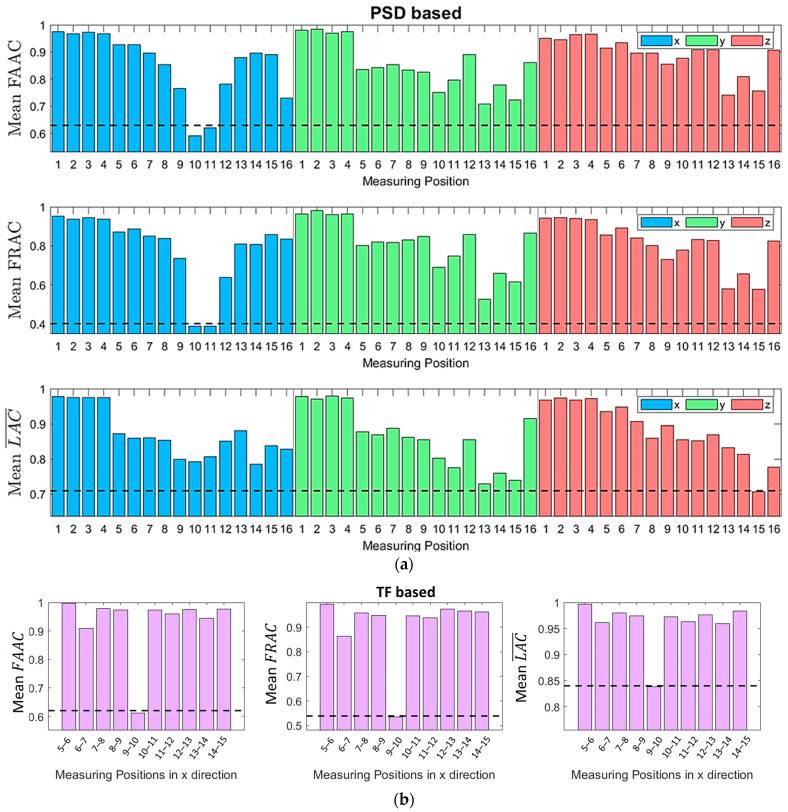
FAAC, FRAC and $\bar{LAC}$ criteria for different measuring positions based on: (**a**) PSD and (**b**) TF. In (**b**), the criteria correspond only to measuring position pairs with good coherence.

The mean values of the FRAC, FAAC, and $\bar{LAC}$ are presented in Figure 8. It is shown that, using the PSD (see Figure 8a), there was agreement only between the FAAC and FRAC, pointing to the 10 and 11 positions in the x direction. On the contrary, with the utilization of the TF (see Figure 8b), all three criteria designated positions 9–10 in the x direction as the optimal measuring locations.

## 3. Damage Detection Methodology

The current section includes a description of the baseline and inspection phases of the evaluated SHM methods. Six methods are employed: two MM-based methods, two PCA-based MM methods, and two FM-based methods. The two MM-based methods [28] are employed using either VAR models for the representation of the structural dynamics [24,25] or TF-ARX models [23]. The methods are abbreviated in these two cases as MM-VAR and MM-TF-ARX, respectively. The PCA-based MM methods, denoted as PCA-MM, represent a refinement or variant of the MM-based methods, introducing PCA parameter reduction before the detection process [28]. In accordance with the MM-based methods, two variants of the PCA-MM methods are examined: the PCA-MM-VAR and the PCA-MM-TF-ARX. Additionally, the FM-based methods [29,30,43,44,45] are employed utilizing either an FP-VAR [45] or an FP-TF-ARX [30,44] model and are, therefore, abbreviated as FM-VAR and FM-TF-ARX respectively. All six methods are trained using pairs of signals that are collected under healthy FOWT operation, with varying EOCs during the baseline phase (see Table 1). It is noted that the varying EOCs characterized by ${\bar{U}}_{hub}$ (see Section 2.3) are measurable in both baseline and inspection phases.

### 3.1. Baseline/Training Phase

During the training phase of the methods, when the FOWT is in the healthy operating state, M pairs of acceleration signal $y\left[t\right]={\left[y_{1}\left[t\right]\;y_{2}\left[t\right]\right]}^{Τ},\;t=1,2,\ldots N$ are acquired from two selected measuring positions. Under each measurement, the ${\bar{U}}_{hub}$ is also recorded by an anemometer and subsequently normalized to the range of $k\in \left[0,1\right]$, resulting in a discrete set of $k_{i}\;\in \left\{k_{1},k_{2},\ldots ,k_{M}\right\}$, each value of which represents the EOCs of the respective measurement. The complete baseline dataset can, therefore, be designated as [43]:

(6) $$Z^{NM}≜\left\{y_{1,k_{i}}\left[t\right],y_{2,k_{i}}\left[t\right]\;\right|\;,\;k_{i}\in \{k_{1},\;k_{2},\;\ldots ,\;k_{M}\}\}$$

#### 3.1.1. Multiple Models (MM)-Based Methods

In the MM-based methods, M models are estimated based on the acquired pairs of signals [27,28]:

(7) $$S_{o}^{M}≜\left\{S_{o,k_{i}}\right|\;k_{i}\in \left\{k_{1},\;k_{2},\;\ldots ,\;k_{M}\right\}\}$$

The VAR and TF-ARX models used in the MM-based methods are concisely presented in the following.

##### VAR Model

A two-response VAR model can be expressed as [46] (pp. 4–5):

(8) $$y\left[t\right]+\sum_{\tau =1}^{n_{a}}A_{\tau }\cdot y\left[t-\tau \right]=w\left[t\right],\;w\left[t\right]\sim iid\;N\left(0,\Sigma_{w}\right),\;E\left\{w\left[t\right]\cdot w^{T}\left[t-\tau \right]\right\}=\Sigma_{w}\cdot \delta \left[\tau \right],$$

where $n_{a}$ designates the model order, $A_{\tau }$ the model [2 × 2] parameter matrix, $E\left\{\cdot \right\}$ statistical expectation, $w\left[t\right]={\left[w_{1}\left[t\right]\;w_{2}\left[t\right]\right]}^{T}$ a [2 × 1] residual vector which ideally is composed of two zero-mean white noise signals following joint identically independent (iid) normal distribution $N\left(0,\Sigma_{w}\right)$ with [2 × 2] covariance matrix $\Sigma_{w}$ and $\delta \left[\tau \right]$ the Kronecker delta ($\delta \left[\tau \right]=1$ for τ=0 and $\delta \left[\tau \right]=0$ for τ≠0). By rearranging the $A_{\tau }$ matrices’ elements into a $\left[4n_{a}\times 1\right]$ vector, the parameter vector [46] (p. 70) is formed:

(9) $$\theta =vec\left({\left[\begin{array}{cccc} A_{1} & A_{2} & \ldots & A_{n_{a}} \end{array}\right]}^{T}\right)$$

where $vec\left(\cdot \right)$ signifies vectorization. An estimation of θ for each individual $k_{i}$ case of the $Z^{NM}$ dataset can be obtained using the ordinary least square (OLS) estimator [46] (pp. 69–72):

(10) $$\hat{\theta }={\left[\Phi^{Τ}\Phi \right]}^{-1}\cdot \left[\Phi^{Τ}y\right]$$

where ^  designates estimate or estimator, $y={\left[y\left[1\right],\;\ldots ,\;y\left[N\right]\right]}^{T}$ the $\left[2N\times 1\right]$ response signal for t=1, …,N and Φ the model’s $\left[2N\times 4n_{a}\right]$ regression matrix including the acceleration signals; see more details about the construction of Φ in [22] (pp. 545–546). Having estimated the parameters of the model, the estimated residual signals (one-step-ahead prediction errors) $w\left[t,\hat{\theta }\right]$ can be obtained based on Equation (8) [46] (p. 94). It is worth emphasizing that $w\left[t,\hat{\theta }\right]$ approximates white noise only if the model can accurately represent the structural dynamics. The residual signals’ uncorrelatedness can, therefore, be assessed to validate the estimated model. The $\left[4n_{a}\times 4n_{a}\right]$ covariance matrix of the estimated parameters is given by [46] (p. 74):

(11) $${\hat{\Sigma }}_{\theta }={\hat{\Sigma }}_{w}⊗{\left[\Phi^{Τ}\Phi \right]}^{-1}$$

where ${\hat{\Sigma }}_{w}$ is the covariance matrix of $w\left[t,\hat{\theta }\right]$ and ⊗ the Kronecker product.

##### TF-ARX Model

A TF-ARX model can be expressed as [22]:

(12) $$y_{2}\left[t\right]+\sum_{\tau =1}^{n_{a}}a_{\tau }\cdot y_{2}\left[t-\tau \right]=\sum_{\tau =0}^{n_{b}}b_{\tau }\cdot y_{1}\left[t-\tau \right]+w\left[t\right],\;w\left[t\right]\sim iid\;N\left(0,\sigma_{w}^{2}\right),\;E\left\{w\left[t\right]{\cdot w}^{T}\left[t-\tau \right]\right\}=\sigma_{w}^{2}\cdot \delta \left[\tau \right]$$

where $n_{a}$, $n_{b}$ the AR and X orders of the model, $a_{\tau }$ and $b_{\tau }$ the AR and X parameters, and $w\left[t\right]$ the model’s residuals, which ideally is a zero-mean white noise signal following an iid normal distribution $N\left(0,\sigma_{w}^{2}\right)$ with variance $\sigma_{w}^{2}$. The model estimation, similar to the VAR model, is performed using OLS (see Equation (10)). To do so, the parameter vector θ $\left[n_{a}+n_{b}+1\times 1\right]$, the regression matrix Φ $\left[{N\times n}_{a}+n_{b}+1\right]$ and the signal vector y $\left[N\times 1\right]$ should be structured appropriately [22] (pp. 203–204). The estimated parameters covariance matrix is, in this case, given by [22] (pp. 551–553):

(13) $${\hat{\Sigma }}_{\theta }={\hat{\sigma }}_{w}^{2}\cdot {\left[\Phi^{Τ}\Phi \right]}^{-1}$$

with ${\hat{\sigma }}_{w}^{2}$, the variance of the estimated residual signal $w\left[t,\hat{\theta }\right]$.

**Figure 9:**
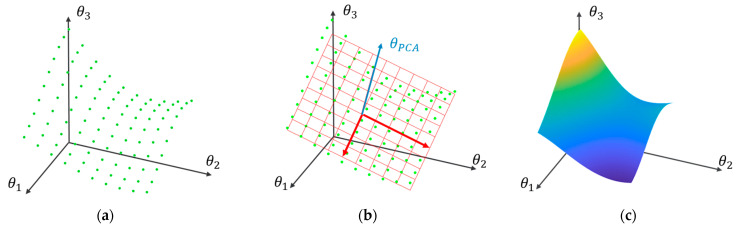
Representation of the structural dynamics: (**a**) discrete subspace representation based on MM; (**b**) subspace dimensionality reduction using PCA; (**c**) continuous subspace representation based on an FP model. Green dots in (**a**,**b**) represent cases of different EOCs. In (**b**), the blue and red axes correspond to principal components, where the blue represents a component with low variability, and the red with arrows represents components with high variability.

By estimating one model for each $k_{i}$, a collection of parameter vectors ${\hat{\theta }}_{k_{i}}$ is obtained. This collection forms a discrete subspace [28] that represents the dynamics of the healthy structure under the different EOCs corresponding to the baseline cases $k_{i}\in \left\{k_{1},k_{2},\ldots ,k_{M}\right\}$ (see example using three model parameters in Figure 9a). This means that during the inspection phase if the k of an unknown case is not included in the ones considered in the baseline phase ($k\notin \left\{k_{1},k_{2},\ldots ,k_{M}\right\}$), the corresponding dynamics of the freshly acquired signals may not be adequately described by any of the baseline models $S_{o}^{M}$, even if the structure is in the healthy state. Therefore, a false alarm will be triggered.

#### 3.1.2. Principal Component Analysis (PCA)-Based Variants of the MM Methods

The idea behind the use of PCA for the improvement of the MM methods is to reduce the dimensionality of the healthy subspace through the exclusion of components that are significantly influenced by EOCs (see Figure 9b). To implement the PCA-MM methods, a set of baseline models $S_{o}^{M}$ is initially estimated, as with the MM-based methods. Then, the cross-case covariance matrix of the models’ parameters is estimated [28]:

(14) $$P=\frac{1}{M}\sum_{i=1}^{M}{\hat{\theta }}_{k_{i}}\cdot {{\hat{\theta }}_{k_{i}}}^{T}$$

and subsequently decomposed as [28]:

(15) $$P=U\Lambda U^{T}$$

with $\Lambda =diag\left(\Lambda_{1},\Lambda_{2},\;\ldots ,\Lambda_{n}\right)$ being a diagonal matrix containing the eigenvalues of matrix P in descending order and U a matrix with the respective eigenvectors. Each eigenvalue indicates the corresponding eigenvector’s contribution to the overall parameter variability. The first q columns of U linked to the higher eigenvalues represent the principal components with the greatest contribution to the estimated parameter’s variability. It is presumed that these components are significantly affected by uncertainties present in the baseline phase, such as those stemming from the varying ${\bar{U}}_{hub}$, and, therefore, they are excluded, while the remaining m components are considered relatively unaffected by those uncertainties and are retained. The number of neglected principal components q, can be determined based on the user-selected fraction of vector variability reduction γ(%) [28]:

(16) $$q=\underset{l}{\min}\left\{l\in \left[1,\;\ldots ,n\right]\;|\;\gamma \leq \frac{\sum_{j=1}^{l}\Lambda_{j}}{\sum_{j=1}^{n}\Lambda_{j}}100\;\%\right\}$$

where n is the size of vector $\hat{\theta }$, which is $n=4n_{a}$ for VAR models and $n=n_{a}+n_{b}+1$ for TF-ARX models. Then, by selecting the m=n−q last columns of U, a matrix $U_{m}$ is formed, which can be used to obtain a reduced version of $\hat{\theta }$**,** which is less affected by uncertainties [28]:

(17) $$\hat{\theta }*=U_{m}^{T}\hat{\theta }$$

(18) $${\hat{\Sigma }}_{\theta *}=U_{m}^{T}{\hat{\Sigma }}_{\theta }U_{m}^{T},$$

where ${\hat{\Sigma }}_{\theta *}$, is the covariance matrix of $\hat{\theta }*$.

A fundamental element of this method is that the cross-case covariance matrix P, contrary to the covariance matrix ${\hat{\Sigma }}_{\theta }$ (given from Equations (11) and (13)), is estimated using $\hat{\theta }$ from different $k_{i}$ cases. Consequently, P incorporates variability arising not only from the stochasticity of the acquired signals but also from the uncertainties present in the baseline phase, such as those due to varying ${\bar{U}}_{hub}$. It should be noted that the selection of high values of γ may result in the neglect of components that are also sensitive to damage. To prevent this from happening, data from the damaged structure may also be used [47]. Otherwise, for unsupervised damage detection, which is considered in the current study, the choice of γ can only be arbitrary, and this is a disadvantage of this method.

#### 3.1.3. Functional Model (FM)-Based Methods

An alternative strategy for utilizing signals from a finite set of discrete EOCs while avoiding errors due to k values not introduced in the training phase is to develop a unified model ($S_{o})$ with parameters that explicitly depend on k, spanning its entire continuous range [29,30,43,44,45] (see Figure 9c). This strategy may be enacted by interpolating the parameters of the models estimated under discrete $k_{i}$ cases ($k_{i}\in \left\{k_{1},\;k_{2},\;\ldots ,\;k_{M}\right\}$), yielding new model parameters for any intermediate k ($k\notin \left\{k_{1},\;k_{2},\;\ldots ,\;k_{M}\right\}$) that may be encountered during inspection. Despite its simplicity, this approach is suboptimal, as the estimation of a model for each $k_{i}$ separately results in an unnecessarily large number of estimated parameters [44]. Moreover, this method overlooks correlations between different sets of signals during the estimation process, leading to an underutilization of available information. To address these drawbacks, the objective of establishing a single global model with functional parameters is accomplished in a statistically optimal manner through the utilization of FP-VAR and FP-TF-ARX models of the following form:

##### FP-VAR Model

The two-response FP-VAR model is formulated as follows [45]:

(19) $$y_{k}\left[t\right]+\sum_{\tau =1}^{n_{a}}A_{\tau }\left(k\right)\cdot y\left[t-\tau \right]=w_{k}\left[t\right],\;w_{k}\left[t\right]\sim iid\;N\left(0,\Sigma_{w}\left(k\right)\right),\;E\left\{w_{k_{l}}\left[t\right]{\cdot w}_{k_{m}}^{T}\left[t-\tau \right]\right\}=\Sigma_{w}\left(k_{l},k_{m}\right)\cdot \delta \left[\tau \right],\;k_{l},k_{m}\in \left[0,1\right]$$

(20) $$A_{\tau }\left(k\right)=\sum_{j}^{p}A_{\tau ,j}\cdot G_{j}\left(k\right)$$

where, $y_{k}\left[t\right]={\left[y_{1,k}\left[t\right]\;y_{2,k}\left[t\right]\right]}^{T}$ the [2 × 1] response vector, $A_{\tau }\left(k\right)$ the [2 × 2] parameter matrix, which is expressed as an explicit function of k, $w_{k}\left[t\right]={\left[w_{1,k}\left[t\right]\;w_{2,k}\left[t\right]\right]}^{T}$ the [2 × 1] serially uncorrelated zero-mean residual (white noise) signal following an iid normal distribution $N\left(0,\Sigma_{w}\left(k\right)\right)$ with covariance matrix $\Sigma_{w}\left(k\right)$, and $\Sigma_{w}\left(k_{l},k_{m}\right)$ the cross-covariance matrix of the white noise signals $w_{k_{l}}\left[t\right]$ and $w_{k_{m}}\left[t\right]$ under $k_{l}$ and $k_{m}$, respectively.

##### FP-TF-ARX Model

The FP-TF-ARX model is formulated as [44]:

(21) $$y_{2,k}\left[t\right]+\sum_{\tau =1}^{n_{a}}a_{\tau }\left(k\right)\cdot y_{2,k}\left[t-\tau \right]=\sum_{\tau =0}^{n_{b}}b_{\tau }\left(k\right)\cdot y_{1,k}\left[t-\tau \right]+w_{k}\left[t\right],\;\;w_{k}\left[t\right]\sim iid\;N\left(0,\sigma_{w}^{2}\left(k\right)\right),E\left\{w_{k_{l}}\left[t\right]\cdot w_{k_{m}}^{T}\left[t-\tau \right]\right\}=\gamma_{k_{l},k_{m}}\cdot \delta \left[\tau \right]$$

(22) $$a_{\tau }\left(k\right)=\sum_{j=1}^{p}a_{\tau ,j}\cdot G_{j}\left(k\right)\;,\;\;b_{\tau }\left(k\right)=\sum_{j=1}^{p}b_{\tau ,j}\cdot G_{j}\left(k\right)$$

where $n_{a}$, $n_{b}$ are the AR and X orders of the model, $a_{\tau }\left(k\right)$ and $b_{\tau }\left(k\right)$ are its AR and X parameters which are explicit functions of k, and $w_{k}\left[t\right]$ a zero-mean, serially uncorrelated residual (white noise) signal which follows an iid normal distribution $N\left(0,\sigma_{w}^{2}\left(k\right)\right)$ with variance $\sigma_{w}^{2}\left(k\right)$ and cross-covariance $\gamma_{k_{l},k_{m}}$.

Equations (20) and (22) indicate that the model parameters are formed through the linear combination of the p mutually independent basis functions $G_{j}\left(k\right)$. These functions establish a functional p-dimensional subspace in which the model parameters reside, with $A_{\tau ,j}$ and $a_{\tau ,j}$, $b_{\tau ,j}$ corresponding to the projection coefficients of the FP-VAR and FP-TF-ARX models, respectively. Any family of orthogonal polynomials may be equivalently used, with the most common choices being the orthogonal Chebyshev and Legendre polynomials.

Equations (19) and (21) state that the residual signals, much like in the simple VAR and ARX models, are serially independent. However, in the FP models, they may be cross-sectionally correlated, meaning that they are correlated across realizations with different k ($k_{l}\neq k_{m}$). While this holds true for FP models in general, in the current application, different k values correspond to entirely distinct time instances. Therefore, the residual signals between different $k_{i}$ should also be uncorrelated.

##### FP Model Estimation

To estimate the FP models’ projection coefficients, Equations (19) and (21) are brought to the form [44,45]:

(23) y=Φ·θ+w

where y is a vector containing the response series for each time instant and all $k_{i}\in \left\{k_{1},k_{2},\ldots ,k_{M}\right\}$ values, Φ the regression matrix with dimensions $\left[\;N\;M\times \left(n_{a}+n_{b}+1\right)p\right]$ for the FP-TF-ARX [44] and $\left[2NM\times 4n_{a}\cdot \;p\right]$ for the FP-VAR [45], w is a vector containing the respective residual signals (for all $k_{i}$), and θ the vector containing the parameters projection coefficients, which are estimated using typical OLS [44,45]:

(24) $$\hat{\theta }={\left(\Phi^{Τ}\Phi \right)}^{-1}\cdot \Phi^{Τ}y$$

For the estimation of the mentioned models, appropriate orders $n_{a},n_{b}$, basis function type and functional subspace dimensionalities p need to be selected. This problem can be solved in two steps. In the first step, the VAR and TF-ARX orders can be selected based on individual pairs of signals under different $k_{i}$. Then, the basis function type and p can be selected, assuming that the same orders $n_{a}$ and $n_{b}$ apply in the FP versions of the models. The system identification process is presented in further detail in Section 4, along with the criteria for the order selection.

For notation’s simplicity, the same symbols $n_{a},\;n_{b},\;p,\theta$ are used in the VAR, TF-ARX, FP-VAR and FP-TF-ARX models without necessarily being equal.

### 3.2. Inspection Phase

In the inspection phase of the methods, a new pair of acceleration signal pairs $y_{u}\left[t\right]={\left[y_{1,u}\left[t\right]\;y_{2,u}\left[t\right]\right]}^{Τ}$ is measured along with k during the FOWT’s current (unknown) operation.

#### 3.2.1. MM-Based and PCA-MM Methods

In the MM-based methods [28], from the collection of the baseline models ($S_{o}^{M}$, see Equation (7)), the model corresponding to the $k_{i}$, which is closest to the current measurement k is selected ($S_{o,k}$). The estimated parameters $\hat{\theta }$ of $S_{o,k}$ are obtained based on the Equation (10) and they are symbolized as ${\hat{\theta }}_{ο}$. Using the acquired signals $y_{u}\left[t\right]$ under the current structural state, a new VAR or TF-ARX model $S_{u,k}$ representing the dynamics of the unknown-state system is estimated with the same order(s) as the baseline models, and the corresponding parameter vector ${\hat{\theta }}_{u}$ is obtained. The characteristic quantity used in this method for detection is the parameter vector of each model. A significant change in the structural dynamics due to damage will also affect the models’ parameters. Therefore, the distance between the parameters of $S_{o,k}$ and $S_{u,k}$ can be used as an indicator for the existence of damage. In the current study, the Mahalanobis distance is used [48]:

(25) $$\delta \hat{\theta }=\sqrt{{\left({\hat{\theta }}_{ο}-{\hat{\theta }}_{u}\;\right)}^{T}{\left(\Sigma_{{\hat{\theta }}_{o}}\right)}^{-1}\left({\hat{\theta }}_{ο}-{\hat{\theta }}_{u}\right)}\;$$

where with covariance matrix $\Sigma_{\;{\hat{\theta }}_{o}}$ of ${\hat{\theta }}_{ο}$ given from Equation (11) for VAR models or Equation (13) for TF-RX models. If, during the selection of the $S_{o,k}$ model, the measured k matches multiple cases from the $S_{o}^{M}$ model collection, a $\delta \hat{\theta }$ is calculated for each, and the minimum among them $\delta {\hat{\theta }}_{min}$ is used. A decision about damage detection is then made if $\delta {\hat{\theta }}_{min}$ is greater than a user-specified threshold $l_{lim}$ [28]:

(26) $$\delta {\hat{\theta }}_{min}\leq l_{lim}\Rightarrow \mathrm{Healthy state}\;\delta {\hat{\theta }}_{min}>l_{lim}\Rightarrow \mathrm{Damaged state}$$

In the PCA-MM methods, the same process is followed, with the difference that $\delta \hat{\theta }$ is calculated using the transformed parameters $\hat{\theta }*$:

(27) $$\delta \hat{\theta }*=\sqrt{{\left(\hat{\theta }\underset{ο}{*}-\hat{\theta }\underset{u}{*}\;\right)}^{T}{\left(\Sigma_{\hat{\theta }\underset{ο}{*}}\right)}^{-1}\left(\hat{\theta }\underset{ο}{*}-\hat{\theta }\underset{u}{*}\right)}$$

with $\hat{\theta }\underset{ο}{*}$ the transformed parameter vector of ${\hat{\theta }}_{ο}$ (see Equation (17)), $\Sigma_{\hat{\theta }\underset{ο}{*}}$ the covariance matrix of $\hat{\theta }\underset{ο}{*}$ (see Equation (18)) and $\hat{\theta }\underset{u}{*}$ the transformed parameter vector of ${\hat{\theta }}_{u}$.

#### 3.2.2. FM-Based Method

In the FM-VAR and FM-TF-ARX methods, the measured k is used on Equations (20) and (22), respectively, for the reparameterization of the global models $S_{o}$. Under the current k value, the FP-VAR and FM-TF-ARX models of Equations (19) and (21) receive the form of the regular VAR and TF-ARX models. These models, representing the healthy state structural dynamics under the current EOCs, are used to make one-step predictions of the acquired signals $y_{u}\left[t\right]$. As mentioned in Section 3.1.1, if the structural dynamics are adequately represented from the model, its residual $w_{u}\left[t,\hat{\theta }\right]$ or $w_{u}\left[t,\hat{\theta }\right]$ should be uncorrelated, while any deviation between the model’s and the actual structural dynamics will lead to nonwhite residuals. Therefore, a decision about the existence of damage can be made by applying a Portmanteau uncorrelatedness test on the model’s residuals. In the case of the FP-VAR model-based method, the metric used for the detection is the statistic $Q_{m}$ of the multivariate portmanteau test [46] (p. 169):

(28) $$Q_{m}=N\sum_{\tau =1}^{h}tr\left(C_{\tau }C_{0}^{-1}C_{\tau }C_{0}^{-1}\right)\;\sim \;\chi_{d_{m}}^{2}$$

where h is the number of lags, $d_{m}$ the degrees of freedom of the distribution which are $d_{m}=4\left(h-n_{a}\right)$ for a [2 × 1] $w_{u}\left[t\right]$ and $C_{\tau }$ the autocovariance matrix:

(29) $$C_{\tau }=\frac{1}{N}\sum_{t=\tau +1}^{T}w_{u}\left[t\right]\;w_{u}^{T}\left[t-\tau \right]$$

For the FP-TF-ARX models-based method, the Ljung–Box test statistic is used [49]:

(30) $$Q=N\left(N+2\right)\overset{h}{\underset{\tau =1}{\sum }}\frac{\rho_{w}\left(\tau \right)}{Ν-\tau }\;\sim \;\chi_{d}^{2}$$

where $\rho_{w}\left(\tau \right)$ is the Autocorrelation Function (ACF) of the residual signal $w_{u}\left[t\right]$ and $d=h-n_{a}-n_{b}$ are the degrees of freedom of the distribution. In both cases, a decision about damage existence can be made based on the $Q_{m}$ and Q theoretical (1−a) critical values:

(31) $$FM-VAR:\;\begin{array}{c} Q_{m}\leq Q_{m,crit}=\chi_{d_{m},1-a}^{2}\Rightarrow \;Healthy\;state\\ Q_{m}>Q_{m,crit}=\chi_{d_{m},1-a}^{2}\Rightarrow \;Damaged\;state \end{array}$$

(32) $$FM-TF-ARX:\;\begin{array}{c} Q\leq Q_{crit}=\chi_{d,\;1-a}^{2}\Rightarrow \;Healthy\;state\\ Q>Q_{crit}=\chi_{d,\;1-a}^{2}\Rightarrow \;Damaged\;state \end{array}$$

**Figure 10:**
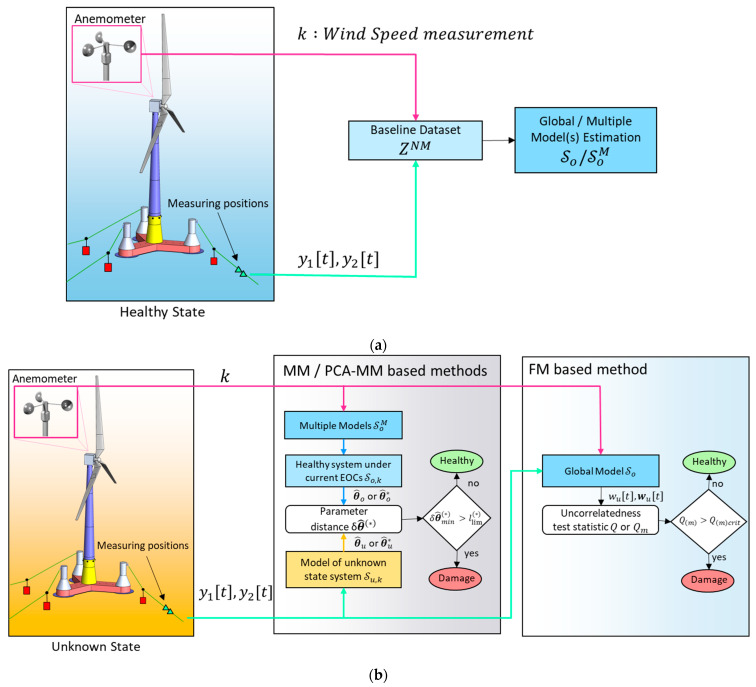
Damage detection framework: (**a**) baseline phase process diagram; (**b**) inspection phase process diagram.

A summary of the general concept of the employed damage detection methods is presented in Figure 10.

## 4. Assessment of Damage Detection Methods through Monte Carlo Simulations

In this section, the performance of the methods outlined in Section 3 is assessed and compared. Specifically, outcomes from the system identification process occurring in the baseline phase of the methods are presented, followed by the detection results for a substantial number of known healthy and damaged cases. For the methods that utilize VAR and FP-VAR models, the used acceleration signals correspond to measuring positions 10 and 11 in the x direction, while for those that utilize TF-ARX and FP-TF-ARX, signals from positions 9 and 10 in the x direction were used—see Figure 3b.

### 4.1. Baseline/Training Phase

The data used in the baseline phase correspond to scenarios of healthy operation, with EOCs characterized by six distinct values of ${\bar{U}}_{hub}$ (see Table 2). For each scenario, ten simulations were conducted using different random seeds, resulting in a baseline dataset $Z^{NM}$ comprising M=60 pairs of signals, each with the length of N=8500 values.

#### 4.1.1. MM-Based Methods

**Figure 11:**
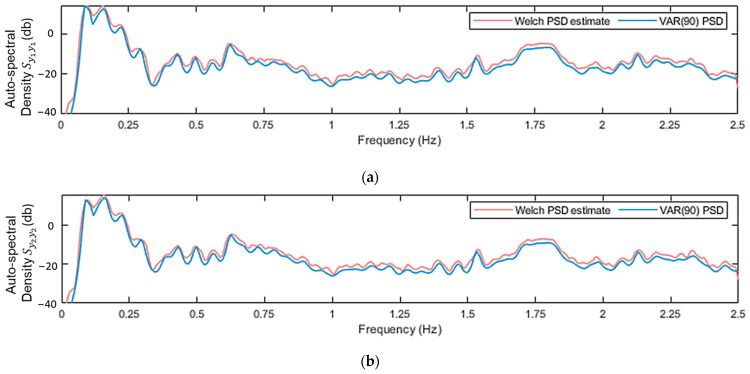
VAR(90) model-based PSD, and nonparametric PSD Welch estimate: (**a**) Autospectral density $S_{y_{1}y_{1}}$; (**b**) Autospectral density $S_{y_{2}y_{2}}$. For the Welch estimate: Hamming Window; window size: 350; $f_{s}\;$= 5 Hz; sample size N=8500; overlap: 95%. Shown for case under ${\bar{U}}_{hub}=10$ m/s.

In the MM-based methods, the primary goal is to determine the most suitable orders for the VAR and TF-ARX models. To achieve this, several criteria were considered, including the Akaike Information Criterion (AIC), the Bayesian Information Criterion (BIC) and the model-based PSD or TF convergence to their nonparametric (Welch-based) counterparts [50]. These criteria were tested for several cases corresponding to different ${\bar{U}}_{hub}$. For the VAR models, a good approximation of the structural dynamics was given with order $n_{a}=90$. The adequacy of this order is demonstrated in Figure 11, where the model’s PSD is compared to its nonparametric estimate for a healthy case with ${\bar{U}}_{hub}=10$ m/s.

**Figure 12:**
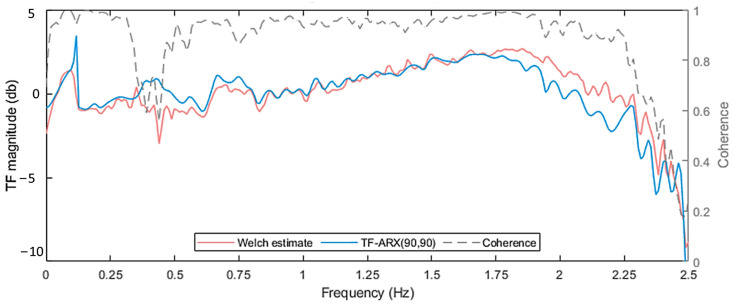
TF-ARX(90,90) model-based TF, Welch-based TF and coherence Welch estimates. For the Welch estimates: Hamming Window; window size: 350; $f_{s}\;$= 5 Hz; sample size N=8500; overlap: 95%. Shown for case under ${\bar{U}}_{hub}=10$ m/s.

**Figure 13:**
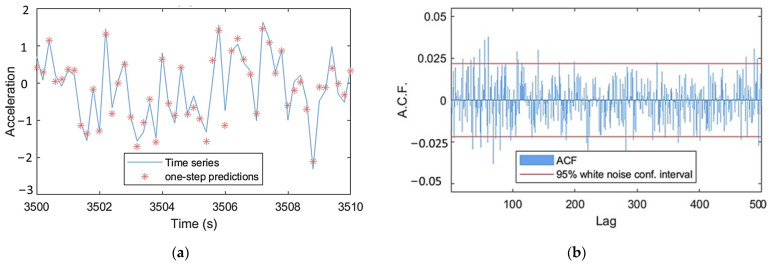
TF-ARX(90,90) model validation: (**a**) one-step predictions and (**b**) residual signals’ Autocorrelation Function (ACF). Shown for healthy case under ${\bar{U}}_{hub}=10$ m/s.

During the TF-ARX identification, notable discrepancies between the models’ and the nonparametric estimates of the TF were observed even for high model orders. These discrepancies were located mainly within the frequency ranges of [0.5–0.6] Hz and [1.7–2.5] Hz and can be associated with the low coherence function at these frequencies (see Figure 12). Nevertheless, as with the VAR models, orders $n_{a}=n_{b}=90$ were selected for the TF-ARX model based on its accurate one-step prediction, see Figure 13.

#### 4.1.2. PCA-MM Methods

**Figure 14:**
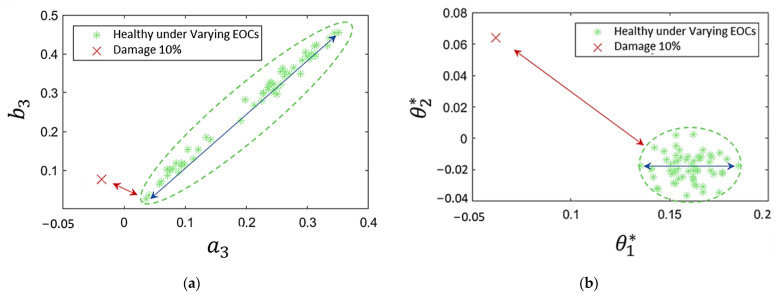
(**a**) TF-ARX parameter scatter plot for healthy and damaged conditions; (**b**) TF-ARX PCA component scatter plot for healthy and damaged conditions. Blue arrows: the maximum distance among all healthy cases of the baseline phase; red arrows: the minimum distance between a damaged case and all healthy cases of the baseline phase.

To reduce the parameter subspace dimensionality with the PCA-MM methods, a decrease in variability of γ=99 % was considered [28], which led to the selection of 135 components for the TF-ARX models and 314 components for the VAR models out of their original 181 and 360 parameters, respectively. The advantage of the PCA-MM over the simpler MM-based methods is demonstrated in Figure 14. In the figure, two scatter plots are compared, representing each condition with two TF-ARX parameters (see Figure 14a) and with two of the selected principal components (see Figure 14b). The performance of each method depends on the parameters’ or principal components’ sensitivity to damage and to EOCs. In Figure 14, the sensitivity to damage is highlighted by the minimum distance between damaged and healthy states (red arrows), while the sensitivity to EOCs is highlighted by the maximum distance between cases of the healthy state (blue lines). It is shown that in the MM-based method (see Figure 14a), the sensitivity to EOCs (blue arrows) is much higher than the sensitivity to damage (red line), while the opposite holds true for the PCA-MM method (see Figure 14b).

#### 4.1.3. FM-Based Method

**Figure 15:**
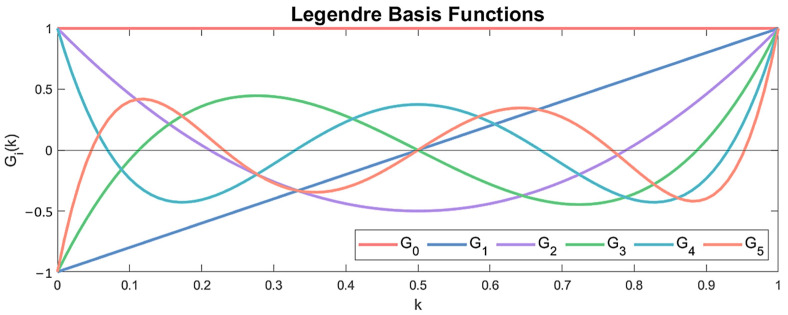
First five Legendre orthogonal polynomials.

The FP model structure identification based on the baseline dataset $Z^{MN}$ involves the $n_{a}$ and $n_{b}$ orders determination, as well as the selection of the appropriate basis function type and dimensionality [43,44]. To simplify the identification problem, the decision was made to employ the same orders as those used for the corresponding VAR and TF-ARX models. For the basis functions, Legendre polynomials of one variable were employed, where each function $G_{i}\left(k\right)$ corresponds to a distinct polynomial order, see Figure 15. The number and combination of Legendre polynomial orders were determined by minimizing the BIC criterion tested for different combinations. The resulting structures for the FP-VAR and FP-TF-ARX models are presented in Table 3.

### 4.2. Inspection Phase

To assess the performance of the six methods, detection outcomes were derived for cases of known healthy and damaged states. The damage scenarios encompass nine distinct levels of stiffness reduction from 10% to 50%, as shown in Table 1. For each state, 11 scenarios of varying EOCs were considered (refer to Table 1), and for each scenario, ten cases were employed, with each case corresponding to a simulation using a different random seed. It is worth emphasizing that cases with ${\bar{U}}_{hub}$ not included in the baseline dataset were also tested in the inspection phase.

As described in Section 3.2, a decision about damage occurrence is determined when the methods’ metrics exceed certain critical thresholds. In the case of MM-based and PCA-MM methods, where the metric is the minimum Mahalanobis distance $\delta {\hat{\theta }}_{min}$ or $\delta {\hat{\theta }}_{min}^{*}$ (see Equations (25)–(27)), the critical limit $l_{lim}$ is user-defined in the baseline phase. For the FM-based methods, the threshold may be set according to the statistical critical value of the portmanteau test or alternatively via a user-selected value. The latter option was adopted in this study, and a user-selected critical limit is employed. For all six methods, the critical limit was determined based on the respective metric’s empirical distribution obtained from the cases of the baseline phase. Specifically, each limit was set at three standard deviations above the mean value of the respective metric.

**Figure 16:**
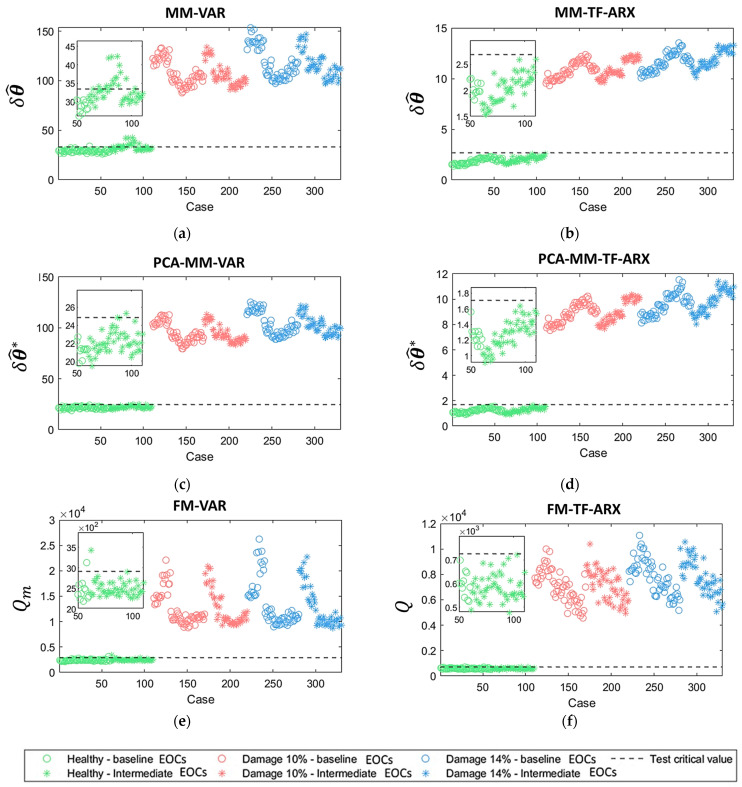
Damage detection results based on the method variations: (**a**) MM-VAR; (**b**) MM-TF-ARX; (**c**) PCAMM-VAR; (**d**) PCA-MM-TF-ARX; (**e**) FM-VAR; (**f**) FM-TF-ARX. Critical values correspond to three standard deviations from the healthy baseline ${\bar{U}}_{hub}$-based metric distributions. Results of (**a**,**c**,**e**) correspond to measuring positions 10–11 and (**b**,**d**,**f**) to 9–10.

The damage detection results of each method are shown in Table 4 in terms of false alarms and correct detections. False alarms refer to instances where known healthy cases are inaccurately categorized as damaged, while correct detections refer to accurately identified damage cases. Inspection results are additionally illustrated in Figure 16 using scatter plots. Each subfigure of Figure 16 corresponds to one of the six employed methods, illustrating the specific metric values for different inspection cases. The inspection cases which have similar ${\bar{U}}_{hub}$ values (7, 8,…, 12 m/s) to the ones used in the baseline phase (see Table 1) are symbolized with circles while cases with intermediate ${\bar{U}}_{hub}$ values (7.4, 8.6,…., 11.4 m/s) with asterisks. The results shown in the figure correspond to the healthy (green) and damage cases with levels 10% (red) and 14% (blue), which represent minor damage. Within Table 4, false alarms corresponding to ${\bar{U}}_{hub}$ values not encountered in the baseline phase are also shown separately.

## 5. Discussion

From the results shown in Table 4 and Figure 16, it is indicated that all six methods were able to detect damage in the synthetic mooring lines with a 100% correct detection rate. This holds true even for damage that is slight enough (10% and 14%) to seemingly have a negligible effect on the structural dynamics (see Figure 4e,f).

A significant rate of false alarms was obtained from the MM-VAR method. As shown in Table 4 and Figure 16a, the majority of these false alarms are associated with cases of ${\bar{U}}_{hub}$ values that are intermediate to the ones found in the baseline phase. When employing the PCA-MM-VAR (see Figure 16c) and FM-VAR methods (see Figure 16e), the false alarm rate dropped significantly, aligning with the intended objective of these methods. Methods MM-TF-ARX (see Figure 16b), PCA-MM-TF-ARX (see Figure 16d) and FM-TF-ARX (see Figure 16f) exhibited excellent results, with zero false alarms. Based solely on these results, neither the FM-based nor the PCA-MM methods seem to have an advantage over the other. However, it’s important to note that the performance of the PCA-MM methods (as discussed in Section 3.1.2) may depend on the selection of the variability reduction γ, which is arbitrary in the absence of damaged-state data in the baseline phase. In light of these observations, it is evident that the FM-TF-ARX method stands out as the most effective among the evaluated methodologies.

The above results pertain to the implementation of the methods using measuring positions 10–11 and 9–10 in the x direction, which were selected in Section 2.6 for the VAR and TF-ARX-based methods, respectively. To assess their sensitivity to the measuring positions, the methods were also employed using positions with a suboptimal ranking based on the FRAC, FAAC and $\bar{LAC}$ criteria. In particular, measurements from positions 5–6 (see Figure 3b) in the x direction were tested. Based on the results shown in Table 5, the methods perform equally well with these measuring positions. This is a significant advantage from a practical point of view, as these positions are located at depths less than 30 m, making sensor installation significantly easier compared to positions 9, 10 and 11, located at depths around 80 m.

Finally, as depicted in all subfigures of Figure 16, there is a clear ‘separation’ between the healthy and damaged states. This implies that defining a critical limit capable of yielding optimal results for any method is feasible, although data from damaged states would be necessary for such determinations.

## 6. Conclusions

The problem of damage detection in FOWT synthetic mooring lines under varying environmental and operational conditions (EOCs) using robust vibration-based SHM methods has been investigated in this study. Six methods were employed based on either Vector Autoregressive (VAR) or Transmittance Function Autoregressive with exogenous input (TF-ARX) models. The methods are founded upon either Multiple Models (MM) or a Functional Model (FM), each serving to represent the structural dynamics under diverse EOCs, utilizing signals from a limited set of baseline data. The PCA variants of the MM-based methods were also explored.

The methods were assessed through 1100 Monte-Carlo simulations using data generated from the finite element model of the OO-Star Wind Floater Semi 10 MW FOWT. The main conclusions follow:

1. All methods show excellent results, being able to detect even slight damage with seemingly negligible impact on the structural dynamics;
2. Both PCA-MM-based methods and FM-based methods reduce the false alarm rate associated with the simpler MM-based methods;
3. The methods utilizing TF-ARX models outperform those using VAR models, achieving perfect detection with zero false alarms;
4. The above methods present excellent results even if sensors are used at randomly selected positions on the mooring line. This facilitates robust SHM as sensors at relatively small depths with simple installation may be employed.

Overall, the results of the current proof-of-concept study indicate that the explored SHM methods are a viable solution for the remote condition monitoring of synthetic mooring lines in FOWTs.

Future plans involve exploring various types and magnitudes of early-stage damage to comprehend the limits of the methods and potentially improve them for damage identification, localization, and quantification. Furthermore, the assessment of the methods using experimental data is also essential, given the unavailability of data from real FOWTs due to their recent installations.

## Figures and Tables

**Table 1:** Baseline and Inspection phase cases (structural state and wind speed) and data description.

	Structural State	Mean Wind Speed (m/s)	No. of Simulations per Scenario	Total No. of Simulations (Sets of Acceleration Signals)
Baseline Phase	Healthy	7, 8, 9, 10, 11, 12	10	60
Inspection Phase	Healthy, Mooring line stiffness reduction: {10, 14, 20, 27, 30, 36, 40, 44, 50} %	7, 7.4, 8, 8.6, 9, 9.5, 10, 10.7, 11, 11.4, 12,	10	1100
$\mathrm{Sampling}\;\mathrm{frequency}:\;f_{s}=5Hz$$,\;\mathrm{Signal}\;\mathrm{bandwidth}:\;\left[0–2.5\right]\;Hz$, Signal length: N=8500 samples.				

**Table 2:** The considered mean wind speeds at hub height ${\bar{U}}_{hub}$ and the corresponding significant wave height $H_{s}$ and peak periods $T_{p}$.

${\bar{U}}_{hub}$ (m/s)	7	7.4	8	8.6	9	9.5	10	10.7	11	11.4	12
$H_{s}$ (m)	1.89	1.95	2.04	2.14	2.21	2.30	2.39	2.53	2.59	2.68	2.81
$T_{p}$ (s)	9.02	9.06	9.13	9.20	9.26	9.32	9.39	9.49	9.54	9.60	9.70

**Table 3:** FP-VAR and FP-TF-ARX model structure identification results.

Model Type	Orders	Basis Function Type	Number of Selected Basis Functions	Polynomial Orders	Number of Projection Coefficients	Samples per Projection Coefficient
FP-VAR	$n_{a}=90$	Legendre polynomials	p=5	0, 1, 2, 3, 4	1800	566.6
FP-TF-ARX	$n_{a}=90,$ $n_{b}=90$		p=4	0, 1, 2, 3	724	1408.9

**Table 4:** Damage detection results overview.

VAR/FP-VAR					
Measuring positions 10–11 for x direction		False Alarms			Correct Detections
	Method	Similar to Baseline ${\bar{U}}_{hub}$	Intermediate to Baseline ${\bar{U}}_{hub}$	Total	Stiffness Reduction Levels 10–50%
	MM-VAR	1/60	17/50	18/110	990/990
	PCA-MM-VAR	0/60	2/50	2/110	990/990
	FM-VAR	1/60	1/50	2/110	990/990
TF-ARX/FP-TF-ARX					
Measuring positions 10–9 in the x direction		False Alarms			Correct Detections
	Method	Similar to Baseline ${\bar{U}}_{hub}$	Intermediate to Baseline ${\bar{U}}_{hub}$	Total	Stiffness Reduction Levels: 10–50%
	MM-TF-ARX	0/60	0/50	0/110	990/990
	PCA-MM-TF-ARX	0/60	0/50	0/110	990/990
	FM-TF-ARX	0/60	0/50	0/110	990/990

**Table 5:** Damage detection results employing acceleration signals from measuring positions 5 and 6 in the x direction.

VAR/FP-VAR					
Measuring positions 6–5 in the x direction		False Alarms			Correct Detections
	Method Variation	Similar to baseline ${\bar{U}}_{hub}$	Intermediate to baseline ${\bar{U}}_{hub}$	Total	Stiffness reduction levels 10–50%
	MM-VAR	0/60	18/50	18/110	990/990
	PCA-MM-VAR	0/60	3/50	3/110	990/990
	FM-VAR	0/60	1/50	1/110	990/990
TF-ARX/FP-TF-ARX					
Measuring positions 6–5 in the x direction		False Alarms			Correct Detections
	Method Variation	Similar to baseline ${\bar{U}}_{hub}$	Intermediate to baseline ${\bar{U}}_{hub}$	Total	Stiffness reduction levels 10–50%
	MM-TF-ARX	0/60	0/50	0/110	990/990
	PCA-MM-TF-ARX	0/60	0/50	0/110	990/990
	FM-TF-ARX	0/60	0/50	0/110	990/990

## Data Availability

The data of this study is available upon request.
